# Poly-Cross-Linked PEI Through Aromatically Conjugated Imine Linkages as a New Class of pH-Responsive Nucleic Acids Packing Cationic Polymers

**DOI:** 10.3389/fphar.2016.00015

**Published:** 2016-02-01

**Authors:** Shun Chen, Tuo Jin

**Affiliations:** Center for BioDelivery Sciences, School of Pharmacy, Shanghai Jiao Tong UniversityShanghai, China

**Keywords:** steric structure, biodegraded polycationic carriers, imine, cross-link, gene delivery

## Abstract

Cationic polyimines polymerized through aromatically conjugated bis-imine linkages and intra-molecular cross-linking were found to be a new class of effective transfection materials for their flexibility in structural optimization, responsiveness to intracellular environment, the ability to facilitate endosome escape and cytosol release of the nucleic acids, as well as self-metabolism. When three phthalaldehydes of different substitution positions were used to polymerize highly branched low-molecular weight polyethylenimine (PEI 1.8K), the product through ortho-phthalimines (named PPOP) showed significantly higher transfection activity than its two tere- and iso-analogs (named PPTP and PPIP). Physicochemical characterization confirmed the similarity of three polyimines in pH-responded degradability, buffer capacity, as well as the size and Zeta potential of the polyplexes formed from the polymers. A mechanistic speculation may be that the ortho-positioned bis-imine linkage of PPOP may only lead to the straight trans-configuration due to steric hindrance, resulting in larger loops of intra-polymer cross-linking and more flexible backbone.

## Introduction

The critical hurdle for turning nucleic acids from therapeutic actives to practical medicines is the lack of therapeutically feasible delivery carriers (Kanasty et al., 2013; Yin et al., 2014). While the ability of viruses to deliver their genetic materials into host cells suggests that the chemical mechanism for designing synthetic carries of nucleic acids is existing in the nature, decades of research efforts including clinic trials of 20 candidates, have yet to reach a practically feasible system (Nguyen and Szoka, 2012; Ginn et al., 2013; Kanasty et al., 2013). To deliver RNA or DNA safely and efficiently into their site of action by systemic administration, a practically feasible synthetic carrier has to be fully functional, structurally simple, and easy to formulate. In fact, none of the systems examined in the clinical trials to date met these criteria. As an attempt to achieve this goal, we recently reported a self-assembled core-shell structured synthetic carrier system consisting a cationic polyplex core to accomplish the intracellular tasks of delivery and a tri-block copolymer membrane to achieve prolonged *in vivo* circulation and target cell recognition (Ge et al., 2015). We hypothesize that designing a core and a shell to address the intra- and intercellular tasks, respectively, will simplify the overall structure and ease the chemical assembly of a synthetic carrier of nucleic acids as compared with conjugating all the functional components to a polymer. This study is one of our continuing efforts in improving the polyplex core.

For intracellular delivery of genetic materials, the polyplex core needs to pack nucleic acids into a nanoparticle, facilitate endosomal escape, release of its cargos in cytosol (for siRNA), or in nucleus (for plasmids), and self-metabolize to non-toxic species (Overly et al., 1995; Pack et al., 2005; Duan et al., 2012). Our previously reported polyimine, polyspermine-4,5-imidazol imine (PSI), showed a highly efficient gene silencing probably due to the unique pKa of its imidazole ring (5.9) for which the nucleic acid-packing polymer degraded to non-toxic monomers in response to the endosomal pH (∼5.8) (Duan et al., 2012). For delivery of DNA plasmids, however, PSI was not as efficient as for siRNA because of its rapid self-degradation and nucleic acid release. For gene delivery, the polyplex forming cationic polymer may need a balanced pH responsiveness and pH resistance to delay the release of the nucleic acids for their approaching to the surface of nucleus. In the present study, therefore, we are trying to establish a spectrum between responsiveness and stability of the aromatic conjugated polyimines by varying the nitrogen-containing heterogeneous rings of different pKa and substitution positions which are involved in the polyimine linkages. Offering a broader choice along a responsiveness-stability spectrum versus a single point is the advance of this study over our previous report. Another objective of this study is, therefore, to examine whether internal cross-linking of the aromatically conjugated polyimines may be used to fine-tune this balance. Our hypothetic rationale is that certain levels of internal cross-linking may retard the overall dissociation the cationic polymer with the same pH responsiveness of the chemical bonds.

As an experimental approach to create different internal cross-linking density, phthalaldehydes of three different substitutions, tere-phthalaldehyde (TP), iso-phthalaldehyde (IP), and ortho-phthaldialdehyde (OP), were used as the linkers to polymerize branched low molecular weight (MW) polyethylene (PEI 1.8 KDa). Although phthalaldehydes are relatively toxic rather than non-toxic heterogeneous aromatic bis-aldehydes, such as imidazole formaldehydes, their three typical substitutions (tera-, iso- and ortho-) and stability provide defined steric differences. The resulted three polymers, poly-cross-linked PEI through tere-phthalimines, iso-phthalimines, and ortho-phthalimines (abbreviated to PPTP, PPIP, and PPOP, respectively) are same stoichiometrically but different sterically. Any differences in their physical/chemical properties and biological behavior should reflect the effect of the steric differences, and elucidating the steric effect may extend our ability in rational design of cationic polymer carriers of nucleic acids.

## Materials and Methods

### Materials

Branched PEI 1.8 and 25 KDa in average MW, were purchased from Sigma–Aldrich. TP, IP and OP were obtained from TCI (Shanghai) Development Co., Ltd. Cellulose membranes for purifying polymeric products of desired MWs (MWCO 10,000 Da) were supplied by Thermo Scientific. All the anhydrous organic solvents were from Sigma–Aldrich, and all the reagents were used without further purification. Plasmid DNA (pDNA) encoding firefly luciferase pGL3-control (Promega) was amplified using EndoFree^TM^ Plasmid Maxi (Qiagen). The sequences of luciferase pGL3-control siRNA were 5′-CUU ACG CUG AGU ACU UCG AdTdT-3′ (sense strand) and 5′-UCG AAG UAC UCA GCG UAA GdTdT-3′ (anti-sense strand).

### Synthesis and Characterization of the Polymers

**FIGURE 1:**
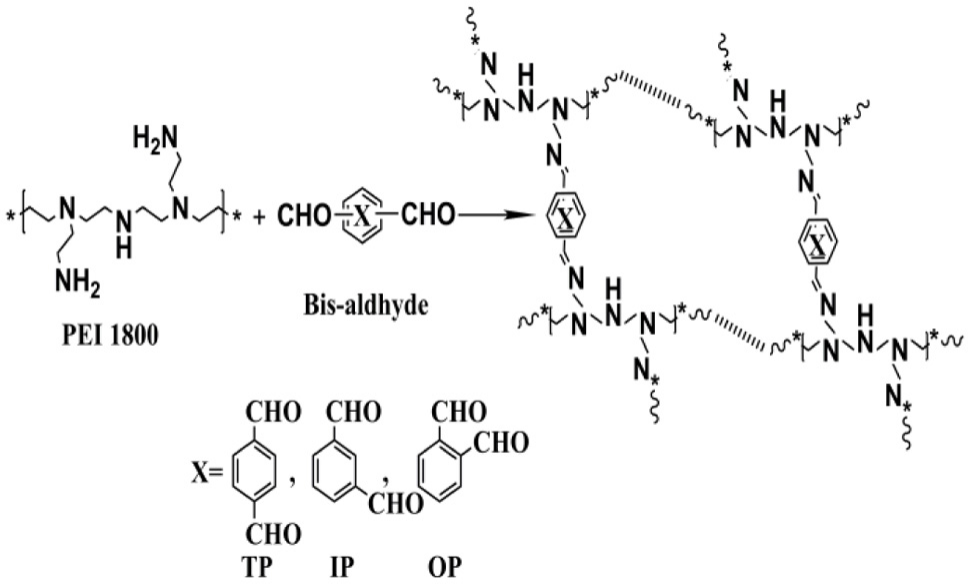
**The reaction scheme of the three polymers based on three steric isomers linkers**.

The designed polyimines were synthesized by condensation of PEI 1.8K with the three linkers (TP, IP, and OP), respectively, as described by the reaction schemes in **Figure 1** according to a reported method (Duan et al., 2012). Briefly, 2 mmol of the linker molecules dissolved in 20 mL anhydrous ethylene dichloride was added dropwise into 1 mmol PEI 1.8K dissolved in 20 mL anhydrous ethylene dichloride under vigorous stirring at room temperature. After 24 h stirring, the solvent was removed by evaporation and the viscous residue was dissolved in deionized water and dialyzed through a cellulose membrane of the MW cutoff of 10,000 Da for additional 24 h. Finally, the polymers of differentiated MWs by dialysis were lyophilized for 2 days prior to storage at -80°C. Formation of the desired polyimines was confirmed using nuclear magnetic resonance (NMR), fourier transform infrared spectoscopy (FT-IR), and gel permeation chromatography (GPC) with polyethylene glycol (PEG) as the standard. The GPC of the polymers were recorded on an Agilent 1260 HPLC system equipped with a refractive index detector (RID) and a thermostatic gel permeation chromatography column PL aquagel-OH. The column temperature was maintained at 40°C and the mobile phase was ddH_2_O at the flow rate of 1 mL/min. Nitrogen content was measured by Elemental Analyzer (Vario-EL Cube, Elementar).

### Degradation and Buffering Capacity of the Polymers

Each of the cationic polyimines was added in deionized water to reach a concentration of 2 mg/mL. Then, the solution was divided to four parts, and pH of the four was adjusted to 5.0, 6.0, 7.4, respectively, by titration with HCl. After the solutions were incubated at 37°C for 3 days, MW changes of the polymers were measured by GPC. The buffering capacity of the polyimines was determined by titrating the polymer solutions from pH 9.0 to pH 4.0. Briefly, 0.1 mg/mL aqueous solution of each of the polyimines were prepared, and titrated with 0.1 M HCl at 25°C. The pH values were recorded with a pH Meter (Mettler Toledo). The buffering capacity was defined as the percentage of amino groups becoming protonated from 7.4 to 5.0 (mimic the pH change from the extracellular environment to the lower pH of the endosome-lysosome system; Overly et al., 1995; Convertine et al., 2010).

### Cytotoxicity of the Polyimines

The cytotoxicity of the polyimines was assessed by cell viability using 3-(4,5-dimethyl-2-thiazolyl)-2,5-diphenyl-2-H-tetrazolium bromide (MTT) assay and hemolytic activity measurement. For MTT assay, each of the three cell lines, COS-7, HeLa and SMMC7721, was incubated with the polymers solutions (dissolved in PBS) of gradually increasing concentrations from 10 to 100 μg/mL for 4 h. Then, 20 μL of MTT (5 mg/mL) solution was added into each well and incubated at 37°C for additional 6 h. Prior to absorbance reading, the medium of each well was replaced with 150 μL of DMSO and the plate was shaking for 10 min at room temperature. Absorbance at 570 nm was measured with Microplate Reader (3M, USA), and reference at 630 nm. The MTT value of untreated cells was considered as 100% cell viability. All transfection and toxicity assays were performed in triplicate. Three linker molecules were investigated at the same process, concentrations from 1 to 20 μg/mL with the same process. Hemolytic activity was utilized to determine membrane lysis of mice erythrocytes by the three polyimines. According to previously reported method, nine BALB/c female mice (5-week-old, weight 20 ± 2 g, obtained from the Institute of Zoology, China Academy of Sciences) were used as the model. The mice were cared for under Specific pathogen Free (SPF) environment in the laboratory animal facility in the school of pharmacy, Shanghai Jiao Tong University. The animal experiments were conducted according to the guidelines approved by the Regulations for the Administration of Affair Concerning Laboratory Animals and in adherence to the National Regulation for Care and Use of Laboratory Animals of China. Mice erythrocytes were separated from fresh citrate-treated blood and washed in PBS by four centrifugation cycles, each at 2000 rpm for 10 min. The red blood cells (RBCs) were diluted in PBS to a final concentration of 2 × 10^8^ erythrocytes/mL. Polymer solutions of different concentrations in PBS (80 μL) and the same volume (80 μL) of RBC suspension were added consequently in to each well of a 96-well plate, followed by incubation at 37°C for 45 min under constant shaking. The cells were also treated with PBS and 1% Triton X-100 as negative and positive control. After centrifugation at 2000 rpm for 10 min, the supernatant in each well was collected and analyzed for the content of hemoglobin released from the erythrocytes using a Microplate Reader (SpectraMax M3, USA) at the wavelength of 450nm. The experiment was repeated three times for calculation of the polymers relative to PBS and Triton X-100.

### Preparation and Characterization of the Polyplexes

Polyplexes were prepared by mixing the polymer solution into a pDNA solution at pre-set nitrogen-to-phosphor (N/P) ratios. Particle size and Zeta potential was determined using dynamic light scattering (DLS) particle sizer (Brookhaven Instruments Corporation 90 Plus). The morphology of polyplexes was imaged under a transmission electron microscope (TEM). Gel electrophoresis, another assay to confirm polyplex formation, was carried out on a 1% (w/v) agarose gel pretreated with 0.5 mg/mL ethidium bromide in 1× Tris-boric acid-EDTA (TBE) buffer at 110 V, and result was imaged using a UV illuminator (Tanon 2500 Gel Image System).

### Transfection *In Vitro*

Transfection experiments were performed on three cell lines, COS-7, HeLa, and SMMC7721, using plasmid pGL3 luciferase as the reporter gene. Cells were cultured to 90% confluence in Dulbecco’s Modified Eagle’s Medium (DMEM, Invitrogen Co., USA) containing 10% FBS (Invitrogen Co., USA) and 1% penicillin/streptomycin (Stock 10,000 U/mL, 10,000 ug/mL, Invitrogen Co., USA). Cells were seeded into 48-well plates at a density of 1 × 10^5^ cells/well 24 h before transfection. When the cells were approximately 80% confluence, washed the cells by PBS twice, and added 250 μL medium without serum at each well. 50 μL polyplexes with different polymer/DNA N/P ratios ranging from 10:1 to 250:1 were gently overlaid into the wells. Each well contains 500 ng pDNA. The plates were incubated at 37°C in a 5% CO_2_ incubator for 4 hours. After incubation, the transfection medium was replaced with 0.5 mL fresh complete medium. The plates were incubated for 48 h under the same conditions as previously. Expression of luciferase was measured according to the instruction. The cells were washed twice with PBS and lysed with lysis buffer (1×, Promega). Cell debris was removed by centrifugation at 12,000 rpm for 3 min (Eppendorf 5810R Centrifuge, Germany) and 20 μL of the supernatant add 20 μL substrate solution (Luciferase Assay System, Promega). The luminescence was measured by Single Tube Luminometer (Berthold Detection Systems GmbH). The total protein concentrations in cell lysates were determined using Micro BCATM Protein Assay Kit (Thermo Scientific Pierce). Luciferase activity was assessed by relative light units (RLUs) per protein concentrations (mg).

### Statistical Analysis

The quantitative data obtained in the experiments of this study were analyzed using the Student *t*-test. For comparative figures, significant differences at the level of ^∗^*p* < 0.05 and very significant differences at the level of ^∗∗^*p* < 0.01.

## Results

### Synthesis and Confirmation of Polyimines

**FIGURE 2:**
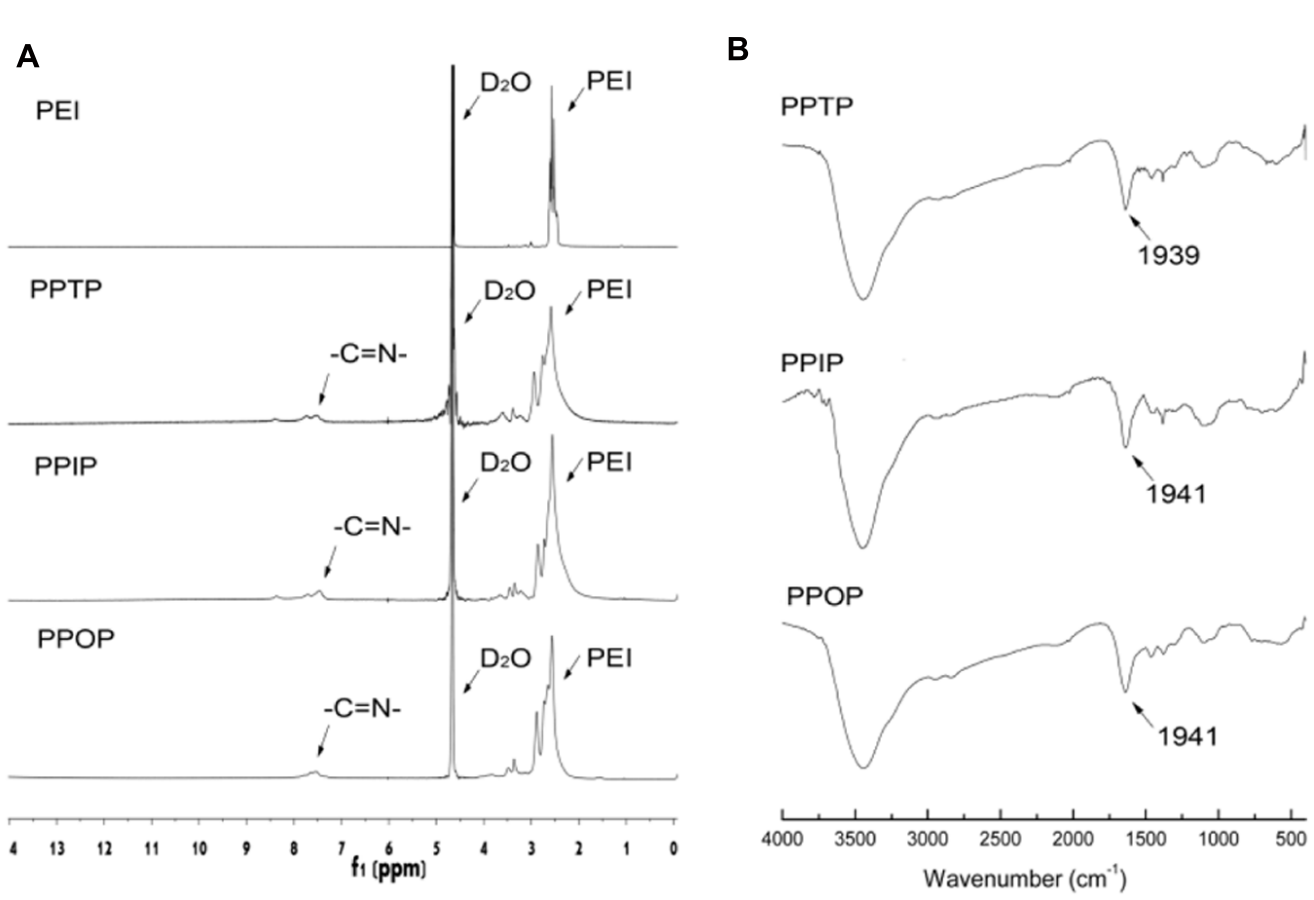
**Structure characterization of the three polymers. (A)** ^1^H-NMR and **(B)** fourier transform infrared spectroscopy (FT-IR).

**FIGURE 3:**
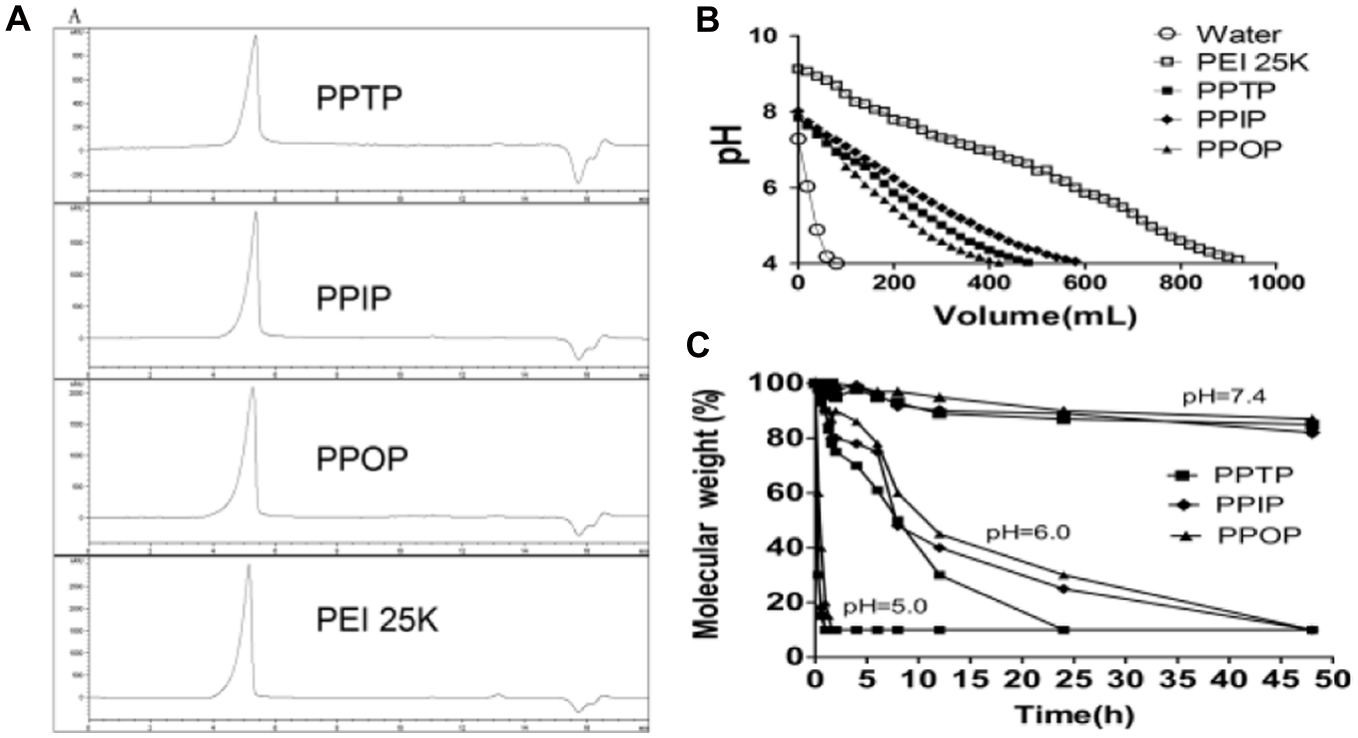
**(A)** Molecular weight (MW) was calculated by GPC methods. **(B)** pH buffering capacity of the polyethylenimine (PEI) derivate from pH 9 to 4 with the same concentration (0.1 mg/ml) and **(C)** Degradation of the three polymers in different pH conditions.

The synthesis of the polyimines was a one-step polymerization as described in **Figure 1**. Formation of three polymers were confirmed by their ^1^H-NMR spectra shown in **Figure 2A**, wherein the chemical shift of the proton over δ10.0 assigned for aldehyde groups disappeared and those at δ7.45–δ7.70 for imine groups appeared instead, indicating the condensation of the aldehydes and primary amines. The formation of the imine linkages were also confirmed by the absorbance at ∼1640 cm^-1^ in the FT-IR spectra for all the three polymers (**Figure 2B**). The average MW of PPTP, PPIP and PPOP were determined by GPC to be 21.5, 20.5, and 21.1 KDa, respectively (**Figure 3A**).

The results of the nitrogen contents were determined by elemental analyzer and shown in **Table 1**. For all the three polymers, nitrogen contents of reduced similarly by one-third. The decrease of nitrogen contents was resulted from the conjugation of the phthaladehyde linkers that contributed mass without nitrogen. Even if the nitrogen content dropping may not be accurate enough quantitatively, the similarity between the three polymers is consistent with their similar MW determined by GPC.

**Table 1:** Nitrogen contents of different cationic polymers.

Polymer	N (%)
Polyethylenimine (PEI 25K)	31.3
PEI 1.8K	31.9
Tere-phthalimines (PPTP)	22.2
Iso-phthalimines (PPIP)	23.1
Ortho-phthalimines (PPOP)	22.3

### Buffering Capability and pH-Responsive Degradation of Polyimines

The buffering capability of the PEI 25K and the three polyimines were determined by acid-base titration. As shown in **Figure 3B**, for the same pH drop, all the three polyimines required less acid than PEI 25K of identical w/v concentration. This titration result is consistent with that of nitrogen content measurement (**Table 1**), by reacting with phthaldialdehydes, the amino content of PEI 1.8K per unit mass dropped significantly as the drop in the amount of titrating acids for the same pH declining. Between the polyimines of similar structure and amino content, however, the difference in buffer capacity was less significant, although that of PPTP and PPIP seemed to be slightly larger than PPOP.

In addition to buffer capacity, the three polyimines showed similar degradation rate at given pH (**Figure 3C**). Dropping of their average MW was accelerated by the decrease in pH. At pH 7.4, the change in average of MW of the polyimines was negligible, but at pH 6.0, similar to that of endosomes, the MW dropped to 50% of its original in 15 h. Further reducing pH to 5.0 resulted in shortened time duration for the MW to reach the 50% of the original from 15 to 0.5 hours (**Figure 3C**).

Compared with our previously reported polyimine, PSI (Duan et al., 2012), the three polyimines in the present study degraded in the same rate at pH 6.0 and 5.0, but much more stable at pH 7.4 (for PSI, the time to reach 50% MW dropping was 48 hours). We also examined the degradability of several polyspermine imines polymerized through aromatically conjugated bis-imine linkages of various pKa, and found no significant differences in the rate of pH-responsive degradation between them (Unpublished data.). These results lead us to suggest that intramolecular cross-linking is an effective way to retard the overall dissociation of the overall backbone of polyimines polymerized through aromatically conjugated imine linkages.

### Cytotoxicity of the Polyimines

**FIGURE 4:**
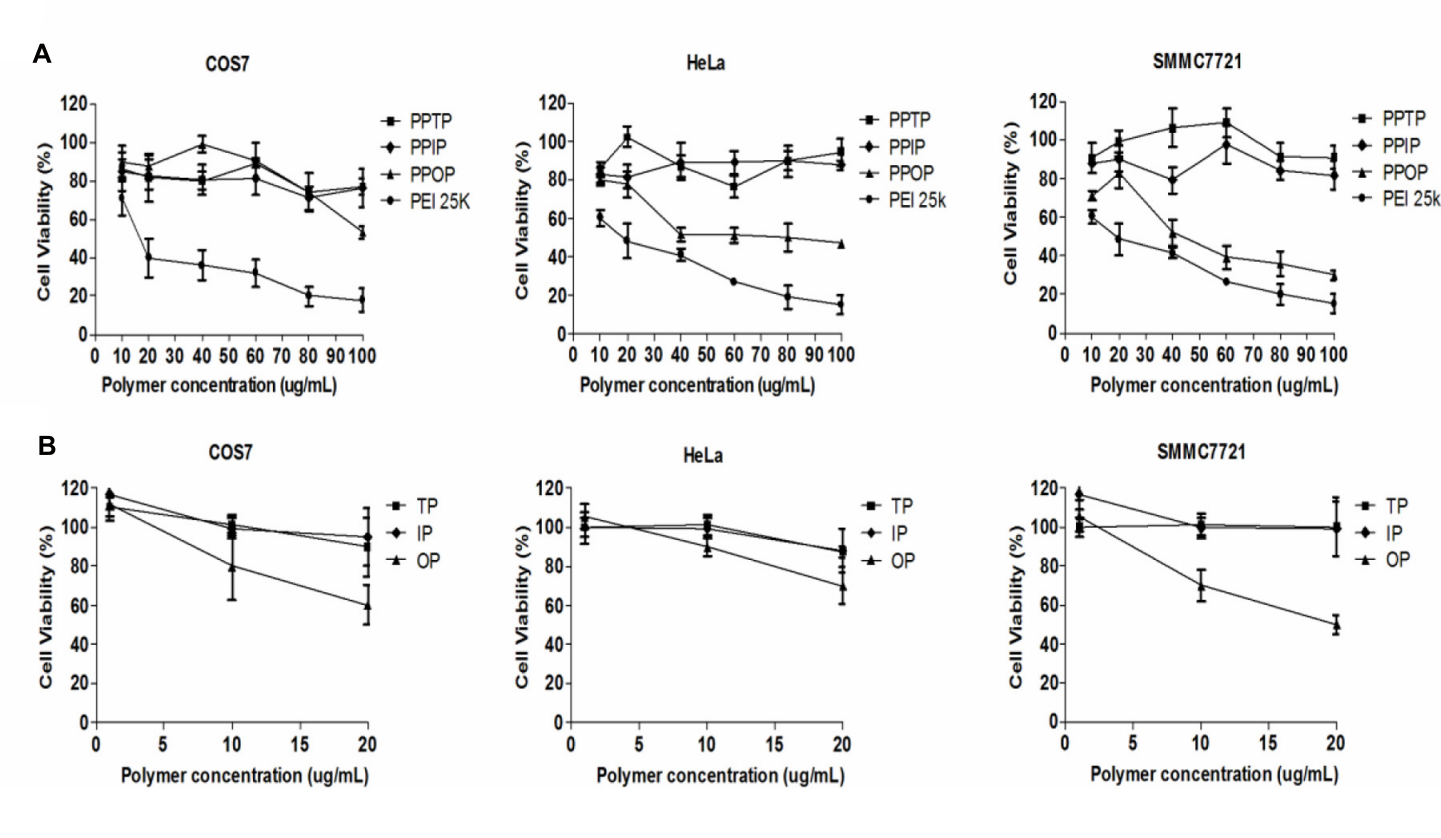
**(A)** Cell viability of three polymers in three cell lines with different concentrations. **(B)** Cell viability of three linker molecules in three cell lines with different concentrations. Considered that nitrogen contents change between PEI 1.8K and three polymers, the linker molecules contents could be about 20% of the polymers after calculation. The concentrations included the degraded linkers concentrations supposed that three polymers from **(A)** had degraded completely.

The cytotoxicity of the three polyimines was examined by the viability of COS7, HeLa, SMMC-7721 cells treated with the polymers of various concentrations. As indicated in **Figure 4A**, although all the three polyimines were less toxic than PEI 25K as expected, PPOP showed significantly higher cytotoxicity than the other two. While some differences appeared between cell lines, the viability of all the cells dropped significantly when the concentration of PPOP was increased, especially for HeLa and SMMC7721 cells (**Figure 4A**). This polymer concentration-depended viability change was not observed for those cells treated with PPTP and PPIP (**Figure 4A**). To elucidate the cause for the severer cytotoxicity of PPOP, the cells were treated with the three difference precursors, TP), IP, and OP of corresponding concentrations, followed by MTT assay for cell viability. The result (**Figure 4B**) indicated that only OP caused cell viability drop among the three phthalaldehydes. However, the orders of the cells in viability changes resulted from the polymers and the phthalaldehydes were not consistent with each other (**Figure 4**). For the treatment by the polymeric PPOP, the viability of COS7 cells remained almost unchanged, but severe viability drop were observed for HeLa and SMMC772 cells. For the treatment by the OP monomer, quite differently, COS7, HeLa, and SMMC7721 cells showed a significant viability drop. Although the OP monomer showed some levels of cytotoxicity, it seems that the decreased cell viability by polymeric PPOP cannot be attributed to the effect of OP.

**FIGURE 5:**
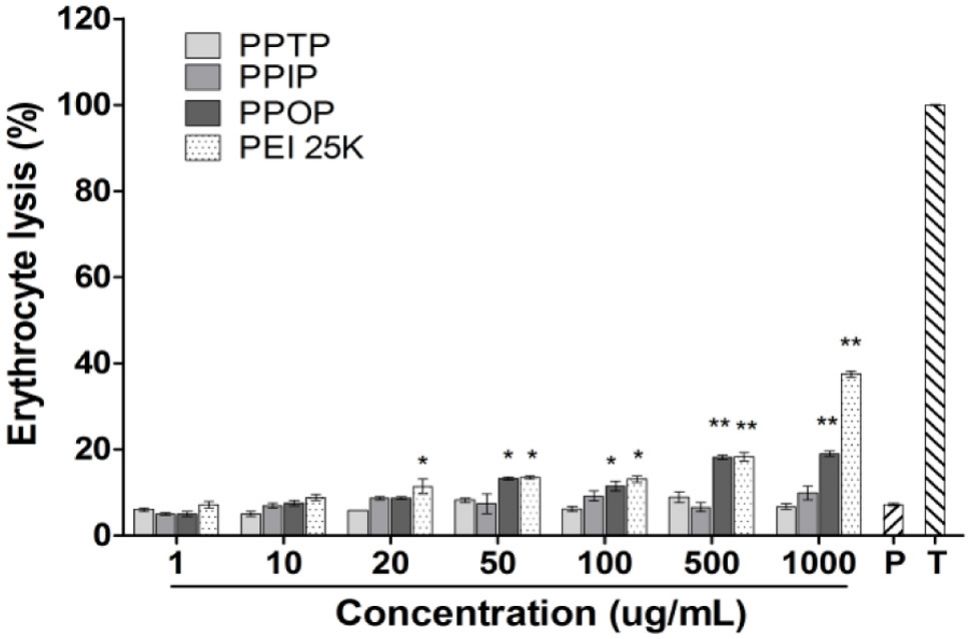
**Hemolytic activity assay**. Mice erythrocytes with different concentrations were incubate with three polymer solutions for 45 min at 37°C and hemoglobin release was quantified at 450 nm. Lysis (100%) refers to Triton X-100 treatment. Results were presented as mean values ± SD of triplicates and the significant was calculated compared with PBS (P = PBS, T = Triton X-100). Ortho-phthalimines (PPOP) and PEI 25K had shown significant (*p* < 0.05) with concentration below 100 ug/mL and highly significant (*p* < 0.01). Tere-phthalimines (PPTP) and iso-phthalimines (PPIP) had no statistical significance.

To further investigate the mechanism of cytotoxicity of the polymers, hemolytic activity of these cationic polymers was measured. Lyse RBCs *in vitro* was also used as an indicator of endosomelytic property (Plank et al., 1994). Cationic polymers above certain concentration may induce hemolytic rupture of red blood cells (RBC) and release of hemoglobin by adsorbing on the anionic cell surface(Rekha and Sharma, 2009). Density of the positive charges and flexibility of the polymer backbone to enable the charged groups to approach their adsorption sites (the negative charge) on the cell surface determine the release rate of hemoglobin (Hong et al., 2006; Lv et al., 2006). As shown in **Figure 5**, when the three polyimines and PEI 25K were added to culture of RBC, with Triton-X100 and PBS buffer as the references, the cells treated with PPOP and PEI 25K showed higher hemoglobin release than those treated with PPTP and PPIP as the polymer concentration increased. In fact, the hemolytic activity of PPTP and PPIP appeared to be same as PBS, indicating negligible damage of the RBC membrane. Assuming the electrostatic interaction between a positively charged polymer and the negatively charged cell membrane causes lytic rupture of the cell, we may conclude at this stage that PPOP of polymeric state was more responsible for the cytotoxicity shown in **Figure 4**. If this argument is true, the fact that the structurally similar polyimines showed different hemolytic activity indicates that steric structure determined the cytotoxicity.

### Polyplexes Formation from Polyimines and DNA

**FIGURE 6:**
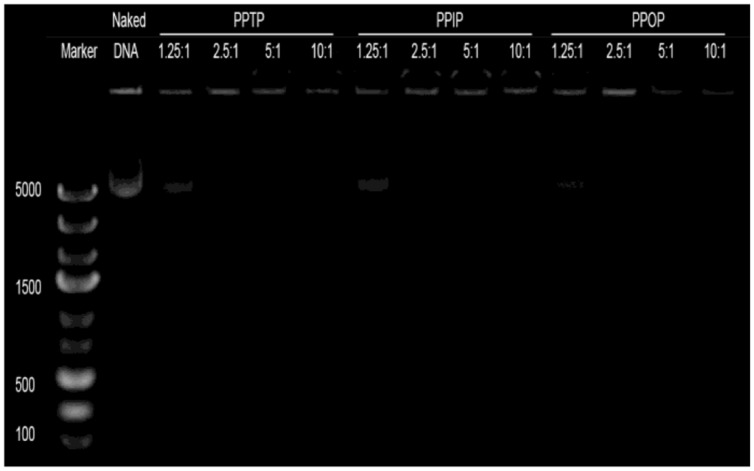
**DNA-binding assay**. The DNA binding abilities of the polymers were examined by agarose electrophoresis with different N/P ratio with DNA.

The formation of polyplexes by mixing DNA with each of the three synthesized polyimines was confirmed using electrophoresis, dynamic light scattering (DLS), and TEM. As a typical method, agarose gel electrophoresis was performed to assay the polyplex formation as well as the DNA condensing capability, the minimal N/P ratio for complete capture of nucleic acids. As shown in **Figure 6**, the electrophoresis band for the tested plasmid DNA became completely invisible when the N/P ratio reached 2.5 for all the three polyimines. This result suggests that under the condition and concentration of the electrophoresis experiment, the three cationic polymers showed the similar DNA packing capability.

**FIGURE 7:**
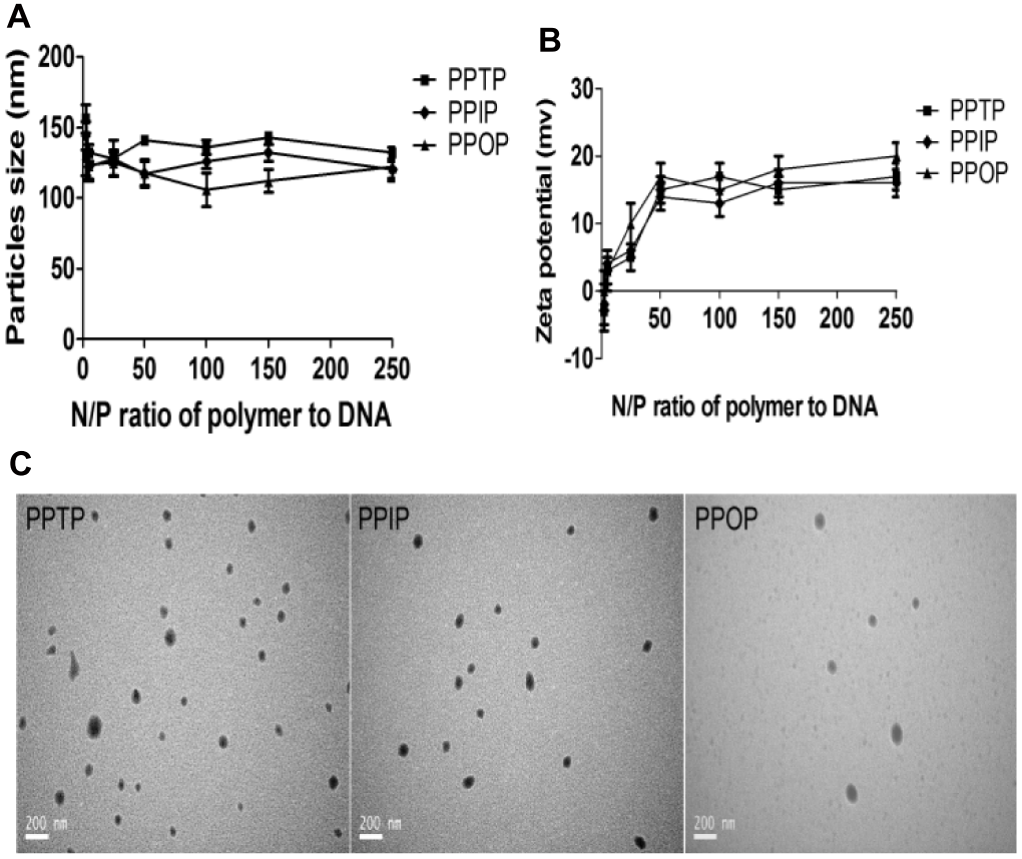
**Physiochemical properties of the polyplexes prepared with the three polymers and plasmid DNAs**. **(A)** Particles size, **(B)** Zeta potential, and **(C)** transmission electron microscope (TEM).

The DLS measurement indicated, on the other hand, that the polyplexes formed from the three polyimines were 120∼150 nm in diameters for all the N/P ratios (**Figure 7A**), and -5 to 0 mV to 15–20 mV in Zeta potential for the polymer/DNA (N/P) ratio was increased from 2.5:1 to 50:1 (**Figure 7B**). The increasing Zeta potential and the invariable diameter of the polyplexes up to the N/P ratio of 50:1 are consistent with the well observed dynamic complexation process that adsorption of cationic polymer result in elevation of Zeta potential, increased in polymer repulsion, and reduce in polyplex mass (Mintzer and Simanek, 2009). Continuous increase in the N/P ratio from 50:1 to 250:1 did not result in further elevation of Zeta potential, suggesting that additional cationic polymer did not involve in the polyplex formation under the polymer concentration of DLS measurement (**Figures 7A,B**). As expected, the polyplex size measure using TEM in dry state was smaller that that determined by DLS in aqueous suspension (**Figure 7C**). The larger size of cationic polyplexes in aqueous suspension may be attributed to the repulsion between the excess of cationic polymer, which consists with our earlier report that cationic polyplexes have plenty of room to shrink in case the position charges are neutralized by oppositely charged surface functionalization components (Ge et al., 2015).

### *In Vitro* Transfection Activity

**FIGURE 8:**
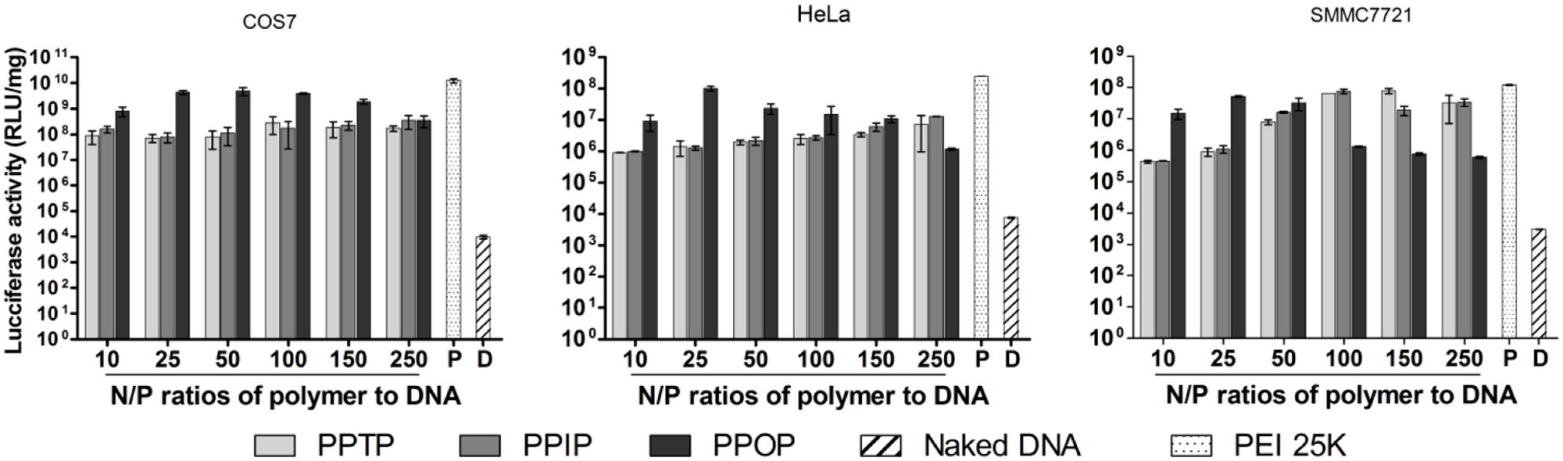
**Transfection efficiency of three polymers in three cell lines with luciferase reported gene**.

The three cationic polyimines were used to pack and transmit luciferase plasmid pGL3 as the reporter gene to each of the cell lines, COS-7, Hela and SMMC7721, followed by determination of luciferase activity. As shown in **Figure 8**, the maximum transfection efficiency for PPOP achieved at w/w ratio of 5:1 (N/P ratio of 40:1) was comparable to that of PEI 25K in all the three cell lines. For PPTP and PPIP, the transfection efficiency was more than one degree of magnitude lower than PPOP and PEI 25K. Although for SMMC7721 cells, PPTP and PPIP reached the similar level transfection, the w/w ratio was as high as 20:1 (N/P ratio of 160:1). Since the three polyimines possess the same MW, buffer capacity, amino group content, and pH-responded degradation rate, the superior transfection activity of PPOP over PPTP and PPIP could only be resulted from their steric differences in structure. We hypothesize that steric hindrance of the ortho-substituted bis-imine linkages in PPOP took only the trans-configuration, which lead to large loops of intra-molecular cross-linking as compared with PPTP and PPIP. And the larger loops allow the overall backbone of the polyimine to be more flexible and accessible to nucleic acids. More discussion will be given in the Section “Discussion”.

Similarly as many cationic polymers, when further increasing the polymer concentration above the optimum polymer to nucleic acid ratio (w/w = 5:1) resulted in activity declining for PPOP (**Figure 8**), suggesting increased cytotoxicity. It is often observed that gene transfection by cationic polymers is facilitated by an optimum polymer-to-DNA ratio to ensure sufficient DNA packaging and tolerable cytotoxicity. Among the three structurally similar polyimines, however, the optimum polymer-to-DNA ratios were considerably different between the structurally similar PPOP and the other two. Both of the transfection assay and cell viability assay support the speculation that PPOP possesses more flexible overall backbone to facilitate the interaction with the anionic charges of DNA as well as cell membranes.

## Discussion

While degradation in response to cellular environment is recognized to be a highly favored criterion in designing cationic polymers for packing nucleic acids, materials in this category are limited to few classes such as poly-β-amino acid esters and polymers linked with di-sulfur bonds (Akinc et al., 2003; Lee and Kim, 2014). Majority of research efforts have been focusing to conjugate functional components for inter-cellular trafficking to cationic polymers. However, multi-steps involved covalent conjugation of functional groups onto a cationic polymer greatly limits the flexibility in designing the nucleic acid packing polymer itself. Our recently invented self-assembly of a tri-block copolymer membrane around each cationic polyplex of any content has provided a very convenient method to protect and functionalize polyplexes at post-polyplexing stage (Ge et al., 2015). Covalent conjugation of inter-cellular delivery components is, therefore, no longer necessary, and design of cationic polymers can be focused on the intracellular tasks only.

By getting rid of the multi-step reactions to link cell targeting components, generally instable cationic polyimines become feasible as pH-responsive nucleic acid packing materials. When conjugated with an aromatic ring, certain levels of acid-resistance of the imine linkages of the polymers may be achieved (Duan et al., 2012). It has been reported that silencing a gene by siRNA or expressing a gene by plasmids demand different intra-cellular functions from a carrier system (Pack et al., 2005). The former is benefited by rapid release with sufficient number of copies of siRNA from the carrier into the cytoplasm of target cells, while the latter requires sustained stability of the polyplexes to facilitate intra-cellular movement approaching the nucleus surface (Lukacs et al., 2000; Lee et al., 2009; Stalder et al., 2013). Endosomes transport cargoes from extracellular environment to the degradative lysosome. It take distinguished time for early endosomes mature to form late endosomes with increasingly acidic mainly through the activity of the V-ATPase (Lafourcade et al., 2008; Stenmark, 2009). For nucleic acids, endosome releasing was very important before endosome–lysosome fusion. Therefore, balancing between the pH-responsiveness and pH-resistance according to the delivery purpose become a factor in designing nucleic acid packing polyimines.

**FIGURE 9:**
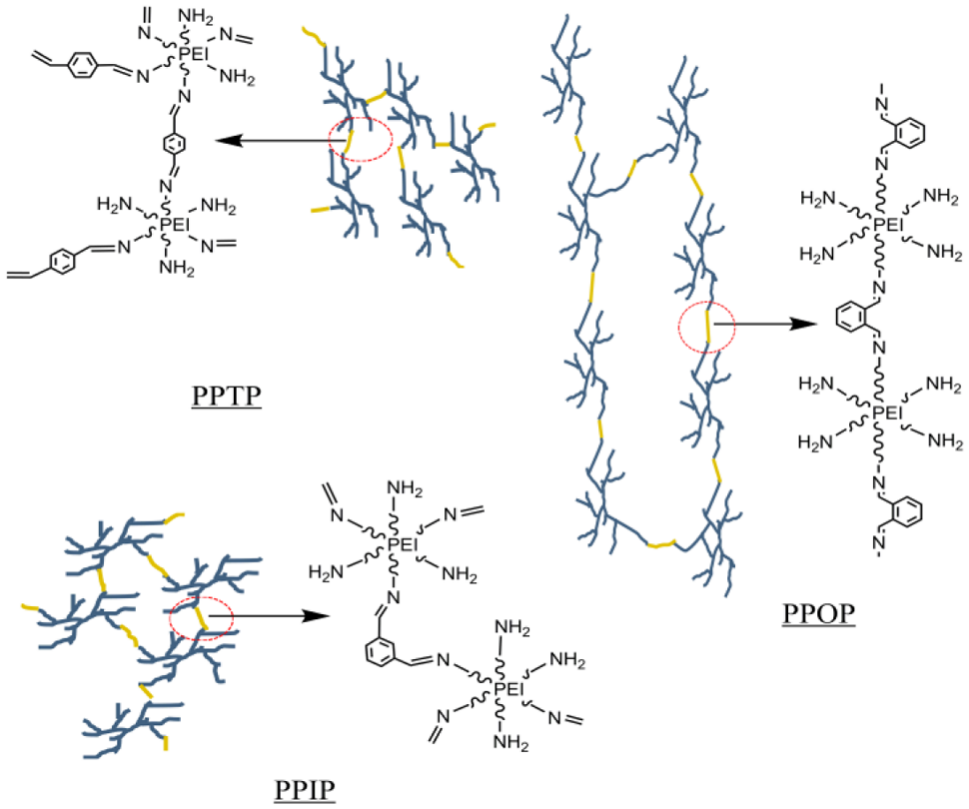
**Chemical structure scheme of PPTP, PPIP, and PPOP**.

For a pH-responsive polyimine, there may be two ways to improve its stability, forming a aromatically conjugated imine linkage which has relative low pKa in terms of proton adsorption, and forming some internal cross-linking to retard overall dissociation of the polymer backbone. In the present study, the second strategy was utilized and highly branched small molecular PEI (PEI 1.8K) was used as the amino group-bearing building block to form the cross-linking loops. At this stage, we recognized that substitution position of the aromatic bis-imine linkage played a critical role in determining gene transfection activity of the polyimine carriers. Since the three poly-phthalimines are the same in chemical bonding, average MW, as well as pH-depended degradation rate (**Figures 2** and **3**), the significant differences in gene transfection efficiency between the ortho-positioned PPOP and the other two may only be attributed to the cross-linking configurations. We therefore hypothesize that the ortho-positioned phthalimine structure can only be formed in the straight trans-configuration due to steric hindrance, resulting in larger cross-linking loops within PPOP (**Figure 9**). For the bis-imine linkage in PPTP and PPIP, the steric availability of both the *trans*- and *cis*-configurations allowed small cross-linking loops to be formed, resulting in firmer overall backbones (**Figure 9**). Since packing RNA or DNA into nano-particulate polyplex is achieved through electrostatic complexation between cationic polymers and anionic nucleic acids, flexibility of the polymer backbone represents better accessibility of the oppositely charged species to each other and more efficient packaging.

For a cationic carrier polymer of a given structure, its gene transfection activity is often associated with its cytotoxicity, because the mechanisms for the polymer to encapsulate anionic nucleic acids and to adsorb on negatively charged cell membranes are the same, electrostatic adhesion. This mechanism applied to the three polyethylene phthalimines of the present study, among which PPOP, the one with relatively flexible backbone, showed superior transfection efficiency and cytotoxicity than the other two analogs, PPTP, and PPIP (**Figures 4**, **5**, and **8**). The higher flexibility of the polymer backbone resulted from larger cross-linking loops in PPOP facilitated the better accessibility of its cationic groups to reach the anionic sites of nucleic acids and cell membranes. For a practical nucleic acid delivering cationic polymer, however, the parallel association between transfection efficiency and cytotoxicity should best be reversed. Such high-transfection efficiency but low toxicity nature was achieved by one of our recently reported polyimines, PSI (Duan et al., 2012). In PSI, the aromatically conjugated bis-imine linkage involved an imidazole ring for which the pKa is 5.9, slightly higher than the endosome pH. For PPOP, this non-toxicity nature may easily be achieved by replacing the OP with the same *cis*-positioned imidazole-4,5-formaldehyde in the polymerization reaction. Replacement by imidazole-4,5-formaldehyde may also resolve another reported cytotoxicity source from OP simultaneously (Morinaga et al., 2010; Iwasawa et al., 2011). As discussed above, such internally cross-linked yet still sufficiently flexible network of PPOP may be an ideal cationic polymer for gene delivery for its enhanced overall backbone stability and nontoxic degrading products (PEI-1800 and imidazole-4,5-formate; Duan et al., 2012).

## Conclusion

Cationic polyimines polymerized through aromatically conjugated bis-imine linkages of various substitution positions is a new class of nucleic acid packing polymers for intracellular delivery characterized with designable pH-responsiveness, stability and backbone flexibility. When a highly branched low MW starter bearing multiple amino groups is allowed to polymerize with aromatic bis-aldehydes, the ortho-substituted aldehyde linker may only form conjugated bis-imine linkages of *trans*-configuration due to steric hindrance. This steric restriction may result in internally cross-linked polymers of relatively larger cross-linking loops as compared with those formed from tera- or iso-substituted aromatic bis-aldehydes. The relatively larger cross-linking loops endow the formed cationic polymer more flexible overall backbone that facilitates the electrostatic condensation of nucleic acids as well as the electrostatic adsorption onto cell membranes, leading to enhanced transfection efficiency and cytotoxicity. Using imidazole-4,5-dialdehyde, an ortho-substituted aromatic bis-aldehyde involving a heterogeneous five-membered ring, to polymerize the highly branched starter should resolve the cytotoxicity and retain the transfection activity of the formed polymer.

## Author Contributions

SC designed the experiments with TJ, carried out the experiments, and drafted the manuscript. TJ initiated this study, involved the experiment design, provided continuous discussion, and finalized the manuscript.

## Conflict of Interest Statement

The authors declare that the research was conducted in the absence of any commercial or financial relationships that could be construed as a potential conflict of interest.
